# The oncological safety of autologous fat grafting: a systematic review and meta-analysis

**DOI:** 10.1186/s12885-022-09485-5

**Published:** 2022-04-11

**Authors:** Rodrigo Goncalves, Bruna Salani Mota, Bruno Sobreira-Lima, Marcos Desidério Ricci, José Maria Soares, Alexandre Mendonça Munhoz, Edmund Chada Baracat, José Roberto Filassi

**Affiliations:** 1 Setor de Mastologia da Disciplina de Ginecologia do Departamento de Obstetrícia e Ginecologia, Hospital das Clínicas da Faculdade de Medicina da Universidade de São Paulo, Avenida Dr. Arnaldo, 251, Secretaria Cirúrgica, 4o andar, São Paulo, SP CEP 01246-000 Brazil; 2 Disciplina de Ginecologia do Departamento de Obstetrícia e Ginecologia, Hospital das Clínicas da Faculdade de Medicina da Universidade de São Paulo, São Paulo, Brazil; 3 Disciplina de Cirurgia Plástica, Hospital das Clínicas da Faculdade de Medicina da Universidade de São Paulo, São Paulo, Brazil; 4 Instituto de Ensino e Pesquisa Hospital Sírio-Libanes, São Paulo, Brazil

**Keywords:** Breast cancer, Breast reconstruction, Lipofilling, Autologous fat grafting

## Abstract

**Objective:**

To present a systematic review of the literature and a meta-analysis evaluating the oncological safety of autologous fat grafting (AFG).

Summary background data: AFG for breast reconstruction presents difficulties during follow-up radiological exams, and the oncological potential of grafted fat is uncertain. Previous studies confirmed that the fatty tissue could be transferred under a good condition suitable would not interfere with mammographic follow-up, although the issue of oncological safety remains.

**Methods:**

We reviewed the literature published until 01/18/2021. The outcomes were overall survival (OS), disease-free survival (DFS), and local recurrence (LR). We included studies that evaluated women with breast cancer who undergone surgery followed by reconstruction with AFG. We synthesized data using the inverse variance method on the log-HR (log of the hazard ratio) scale for time-to-event outcomes using RevMan. We assessed heterogeneity using the Chi^2^ and I^2^ statistics.

**Results:**

Fifteen studies evaluating 8541 participants were included. The hazard ratios (HR) could be extracted from four studies, and there was no difference in OS between the AFG group and control (HR 0.9, 95% CI 0.53 to 1.54, *p* = 0.71, I^2^ = 58%, moderate certainty evidence), and publication bias was not detected. The HR for DFS could be extracted from six studies, and there was no difference between the AFG group and control (HR 1.01, 95% CI 0.73 to 1.38, *p* = 0.96, I^2^ = 0%, moderate certainty evidence). The HR for LR could be extracted from ten studies, and there was no difference between the AFG group and control (HR 0.86, 95% CI 0.66 to 1.12, *p* = 0.43, I^2^ = 1%, moderate certainty evidence).

**Conclusion:**

According to the current evidence, AFG is a safe technique of breast reconstruction for patients that have undergone BC surgery and did not affect OS, DFS, or LR.

**Supplementary Information:**

The online version contains supplementary material available at 10.1186/s12885-022-09485-5.

## Introduction

Autologous fat grafting (AFG) in the breast, to improve its volume and form, was first described at the end of the last century [1]. This technique has been used since the beginning of liposuction, under the term lipofilling. AFG, for breast reconstruction, presents difficulties during follow-up in radiological exams, and the oncological potential of grafted fat is uncertain. These issues lead to a recommendation from the American Society Of Plastic and Reconstructive Surgeons, contraindicating the technique in the breast’s aesthetic and reconstructive plastic surgery in 1987 [2]. However, the work of Coleman and Saboeiro in 2007 confirmed that the fatty tissue could be transferred under suitable conditions, provided that a rigorous preparation and transfer protocol is respected and would not interfere with mammographic follow-up, although the issue of oncological safety remained controversial [3].

Additionally, AFG complications include calcifications, fat necrosis, and cyst formation, which can potentially restrict the early diagnosis of breast cancer and the follow-up of patients with a history of breast cancer [4]. Recently, AFG has been indicated for conservative and radical surgery reconstruction following breast cancer and after or combined with risk-reduction procedures. In conservative treatment, it allows for correcting a defect, a retractile scar, and insufficiency of breast volume. In radical surgery, AFG may complement the reconstruction, and it can be used either before or after radiation treatment to correct breast implant exposure,e among other defects [5, 6]. Recent publications have shown that AFG can be used as the only alternative to breast reconstruction in patients with breasts of small volumes [7]. AFG is helpful in the various stages of breast reconstruction, correcting contours, ending with a more natural appearance of the breast in eligible patients. Despite the current widespread indication and utility of AFG, some questions are related to the AFG technique and its oncological safety.

Currently, there is no evidence to support a specific technique of AFG as a gold standard because of the absence of well-designed prospective studies [8].

The uncertainty surrounding AFG safety is due to adipose-derived stem cells (ASC) in angiogenesis, tissue regeneration, inflammation, and wound healing. Translational studies on this subject resulted in conflicting evidence. Goto et al. [9] demonstrated that culture of patient-derived-xenograft cells with ASC promoted the growth of tumors, increasing their volumes and burden in immunodeficient mice, mediated by ASC-secreted adipsin. Gebremeskel et al. [10] however, showed that although culturing breast cancer cells in ASC-conditioned media caused an increase in cell proliferation, the same effect was not observed when the cells were cultured in fat graft-conditioned media. Tsuji et al. [11] and Silva et al. [12] found similar results when MDA-MB-231 or MCF-7 cancer cells were mixed with human fat grafts and injected directly into mice. These authors found that mice receiving fat grafting presented lower tumor volumes, possibly having a protective effect on tumor growth.

As treatment recommendations and surgical approaches evolve, decisional conflict may arise when patients with breast cancer (BC) diagnosis face the need to choose a management option, including AFG. In addition, various clinical studies have been published investigating the outcome of AFG as a reconstructive technique following breast cancer surgery [4]. However, a significant part of these studies has been inconclusive and with a lower power of evidence. Most of the current clinical evidence is limited by the retrospective nature of the data, small sample sizes, and relatively short follow-up periods [13].

Thus, to address knowledge gaps regarding the oncological safety of AFG in partial and total breast reconstruction, the present meta-analysis was performed.

## Methods

We conducted a systematic review and a meta-analysis to evaluate the oncological safety of AFG after breast cancer surgery. We thoroughly reviewed the peer-reviewed literature on the subject published until 01/18/2021. The analyzed outcomes were overall survival (OS), disease-free survival (DFS), and local recurrence (LR).

### Inclusion and exclusion criteria

We included randomized controlled trials, cohort studies, case-control studies which evaluated women with a breast cancer diagnosis who underwent surgery followed by immediate or delayed breast reconstruction with AFG, with control groups in which breast reconstruction did not include AFG.

Case series, duplicate papers, duplicate data, and manuscripts without original data (e.g., comments, reviews, case reports, and technical descriptions) were excluded.

### Search strategy

This review was performed following the PRISMA guidelines (Preferred Reporting Items for Systematic Reviews and Meta-analyses) [14]. We performed searches in the electronic databases of Medline (via PubMed), EMBASE (via OVID), LILACS (Latin American and Caribbean Health Sciences Literature and Cochrane Library using combinations of search terms for autologous fat grafting and breast cancer. Two reviewers independently assessed all titles and abstracts for possible inclusion. All disagreements were resolved via consensus discussion with a third researcher. There was no language restriction. The search strategies for each database can be found in the Additional file 1.

### Data extraction

The following data were retrieved from the studies independently by two reviewers: publication details, study design, study setting, inclusion and exclusion criteria, methods used to control for confounders, characteristics of patients (age, stage, follow up, adjuvant treatment), details of the intervention, outcome measures and withdrawals. All data were obtained from the published results and are summarized in Table 1.

**Table 1:** Main characteristics of the included studies. The oncological safety of autologous fat grafting: a systematic review and meta-analysis

Author	Fertsch [15]	Cohen [16]	Calabrese [17]		Cogliandro [18]	Khan [19]	Krastev [20]	Kronowitz [21]
Type of study	Case-control	Cohort	Cohort		Cohort	Case-control	Cohort	Cohort
Year	2017	2017	2018		2017	2017	2019	2015
Number of patients	200	829	233		70	71	587	2364
Number of cases	100	248	105		46	32	300	1024
Age								
AFG	49.6	47,8/48,1^a^	48,8/50,3^b^		41^c^	49	48.1	47,7/45,8^a^
No AFG	50.7	52,6/49^a^	47,7		41^c^	54	49.4	46,5
Follow up (months)								
AFG	72.5	45,6/42,5^a^	84/75^b^		30^c^	36	112	59,6/73,5^a^
No AFG	76.5	38,8/37,6^a^	72		30^c^	36	103	43.8
Stage								
Stage 0 - AFG	9	51/NA^a^	5/9^b^		NA	NA	39	174/16^a^
Stage 0 - no AFG	9	83/NA^a^	6		NA	NA	40	115
Stage 1 - AFG	NA	55/NA^a^	16/38^b^		NA	NA	99	266/14^a^
Stage 1 no AFG	NA	149/NA^a^	26		NA	NA	102	208
Stage 2 AFG	NA	46/NA^a^	20/17^b^		NA	NA	114	199/23^a^
Stage 2 no AFG	NA	143/NA^a^	32		NA	NA	107	245
Stage 3 AFG	NA	10/NA^a^	0		NA	NA	48	65/6^a^
Stage 3 no AFG	NA	39/NA^a^	0		NA	NA	51	92
Prophylactic surgery	No	No/Yes	No		No	No	No	No/Yes
Breast Reconstruction Type	DIEP	Tissue expander or Autologous or Implant	Tissue expander + Implant		Implant	NA	NA	NA
AFG technique	Coleman	Coleman	Coleman + SVF		Coleman	Coleman	Coleman	NA
Author	**Masia** [22]	**Stumpf** [23]	**Sorrentino** [24]	**Silva-Vergara** [25]	**Seth** [5]	**Petit DCIS** [26]	**Petit Invasive** [27]	**Mazur** [28]
Type of study	Cohort	Cohort	Cohort	Cohort	Cohort	Case-control	Case-control	Case-control
Year	2015	2017	2019	2017	2012	2013	2012	2018
Number of patients	214	194	830	615	886	177	963	308
Number of cases	107	27	233	205	69	59	321	56
Age								
AFG	49.2	53.6	49.4	49.1	49.4	46	45	NA
No AFG	48.9	56	51	50	48	47	46	NA
Follow up (months)								
AFG	89	36	74.1	88.7	43.6	63	56	36
No AFG	120	36	63.8	86.8	42.1	66	57	NA
Stage								
Stage 0 - AFG	61	0	31	0	17	59	37	NA
Stage 0 - no AFG	69	0	71	0	176	118	74	NA
Stage 1 - AFG	23	7	94	109	23	0	174	NA
Stage 1 no AFG	26	78	289	237	212	0	348	NA
Stage 2 AFG	14	20	71	79	23	0	86	NA
Stage 2 no AFG	5	89	178	135	288	0	172	NA
Stage 3 AFG	5	0	37	11	4	0	24	NA
Stage 3 no AFG	2	0	58	23	87	0	48	NA
Prophylactic surgery	No	No	No	No	No	No	No	NA
Breast Reconstruction Type	DIEP, SIEA, SGAP, IGAP, TAP	Breast conserving surgery plus AFG	NA	NA	NA	NA	NA	NA
AFG technique	Coleman	Coleman	Coleman	Coleman	Coleman	Coleman	NA	Coleman

*AFG* Autologous fat grafting, *DIEP* deep inferior epigastric artery perforator flap, *IGAP* inferior gluteal artery perforator flap, *NA* not available, *SGAP* superior gluteal artery perforator flap, *SIEA* superficial inferior epigastric artery flap, *SVF* stromal vascular fraction, *TAP* thoracodorsal artery perforator flap

^a^ in Cohen et al. and Kronowitz et at, the authors performed AFG for patients that undergone cancer surgery and prophylactic surgery. In these studies, the number on the left refers to the patients that undergone cancer surgery and the number on the right refers to the patients that undergone prophylactic surgery

^b^ in Calabrese et al., the authors employed two modalities of AFG. The number on the left refers to the patients that undergone AFG with adipose tissue enriched with stem cells from the stromal vascular fraction. The number on the right refers to classic Coleman AFG technique

^c^ in Cogliandro et al., the authors do not present the age and follow-up according to study groups; they only present the mean age and mean follow-up for the whole population

### Assessment of risk of bias of the included studies

Two independent reviewers assessed the methodological quality of the studies using the Downs and Black instrument [29]. This quality assessment checklist comprises 27 questions, with a maximum possible score of 28 points for randomized studies and 25 points for non-randomized studies. The reviewers assessed the methodological quality of each study and the risk of bias for the following domains: reporting bias (10 items), external validity bias (3 items), internal validity bias (7 items), confounding bias (6 items), and power of the studies (1 item). We gave scores of 0 or 1 for each risk of bias domain and the associated specific questions, except for one item related to the analysis of the distribution of confounders, which was scored 0, 1, or 2. Finally, the overall quality of evidence for each study was rated depending on the final score: excellent (score 26 to 28), good (score 20 to 25), fair (score 15 to 19), or poor (< 14).

### Statistical analysis

#### Data synthesis

We synthesized data using RevMan [30]. The appropriate unit of analysis was the individual participant rather than the breast, surgical unit, hospital, or center. We combined data using the inverse variance method on the log-HR scale for time-to-event outcomes and the log-RR scale for dichotomous outcomes. When the data were too diverse to permit the combination of effect sizes in a meaningful or valid manner, we presented the results of individual studies in a table and graphical formats and used a narrative approach to summarize the data.

#### Measures of treatment effect

We reported time-to-event outcomes (e.g., OS, DFS, and LR) as hazard ratios (HRs) with 95% confidence intervals (CIs). Where only published survival curves were available, hazard ratios were calculated using the method of Parmar et al. [31], which assumes that censoring occurs uniformly between the minimum and maximum follow-up times reported in the study. Where numbers at risk were reported in Kaplan-Meier curves, the method of Williamson and Tierney et al. was used [32, 33]. We reported dichotomous outcomes as risk ratios (RRs). We pooled the data for meta-analysis using the pooled log-RR, when appropriate. We reported continuous outcomes (e.g., quality of life) as mean differences (MDs) with 95% CIs.

#### Assessment of reporting biases, missing data, and heterogeneity

We contacted study authors to establish a complete data set or reasons for the non-reporting of specific outcomes. When ten or more studies were in meta-analyses, we would perform a funnel plot and egger’s test to investigate publication bias [34]. If publication bias was detected, we conducted a sensitivity analysis, excluding the potential source of bias.

When data were missing or unsuitable for analysis (e.g., intention-to-treat is not used), we contacted the study authors to request further information. When data were missing to the extent that the study cannot be included in the meta-analysis and attempts to retrieve data have been exhausted, we presented the results in the review and discussed the context of the findings.

If appropriate, we assessed the presence of statistical heterogeneity using the Chi^2^ statistic, and we investigated its extension by using the I^2^ statistic, which estimates the percentage of total variation across studies due to heterogeneity rather than chance. An I^2^ value of 30 to 60% may represent moderate heterogeneity, and values greater than 50% may be considered to show substantial heterogeneity [35].

### GRADE and ‘summary of findings’

We created a ‘Summary of findings table for the main outcomes for the comparison of AFG versus control. Two authors (BSM and RG) independently assessed the certainty of the evidence using the GRADE framework. We used the five GRADE criteria (study limitations, consistency of effect, imprecision, indirectness, and publication bias) to assess the certainty of a body of evidence and reported this certainty as either high, moderate, low, or very low. We considered the following criteria for upgrading the certainty of evidence, if appropriate: large effect and dose-response gradient. We used the methods and recommendations described in Sections 8.5 and 8.7 and Chapters 11 and 12 of the Cochrane Handbook for Systematic Reviews of Interventions [36–38].

We used GRADEpro software [39, 40] (available at https://gradepro.org) to prepare the ‘Summary of findings table. We justified all decisions to downgrade or upgrade the certainty of the evidence using footnotes and made comments to aid the reader’s understanding of the review where necessary. The following outcomes were selected for the Summary of findings table: overall survival, disease-free survival, and local recurrence.

To calculate the absolute risk for the control group for time-to-event outcomes, we estimated the event rate at a specific time point (i.e., the five-year time point for both overall survival and disease-free survival) from the Kaplan-Meier curves or based upon an average of the estimates from studies. We entered these estimated values in GRADEpro GDT software, which automatically populated the corresponding absolute risks for the intervention group at the five-year time point.

## Results

### Study characteristics

**Fig. 1:**
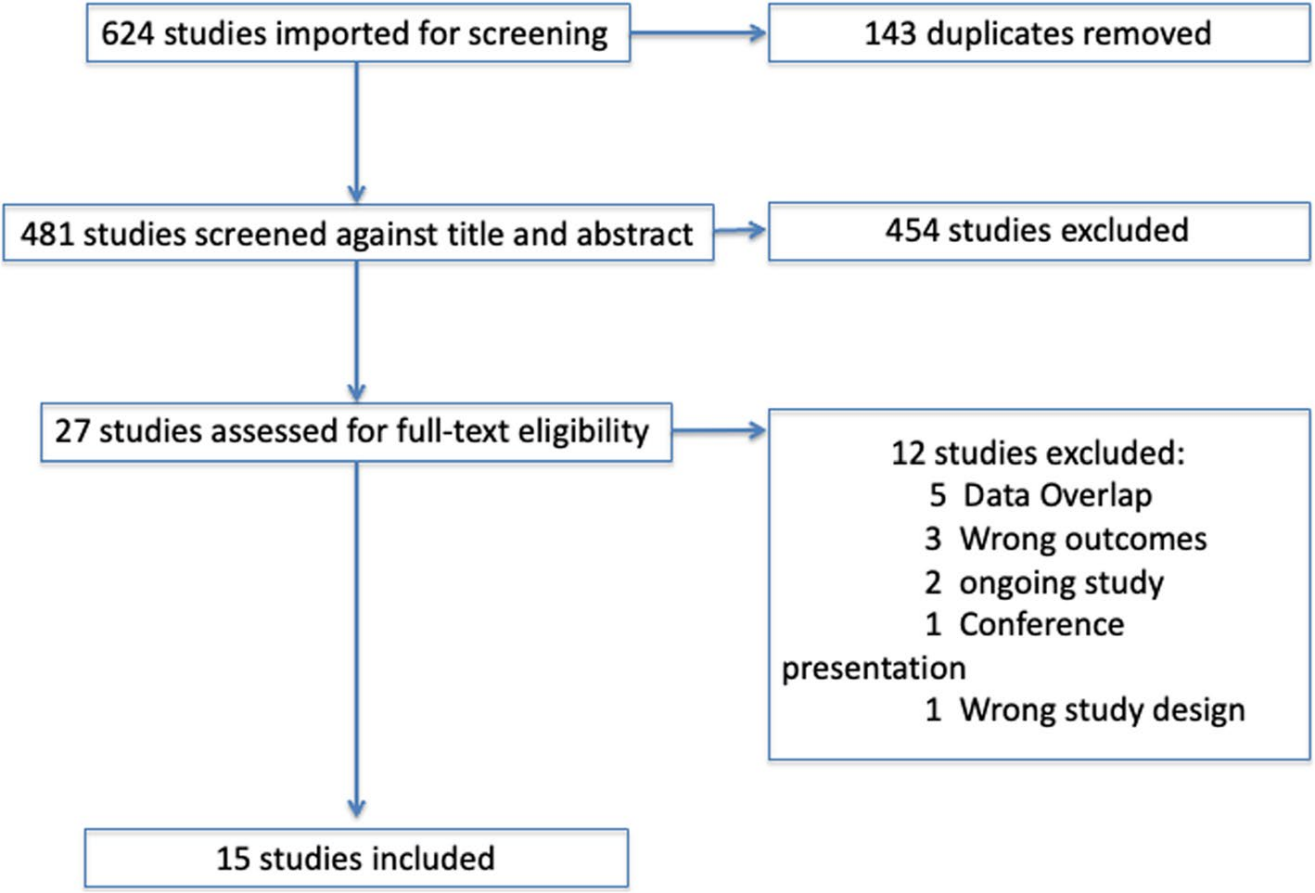
Flowchart of the study selection process according to PRISMA guidelines

Based on our search strategy, 624 references were identified and screened. After removing duplicates, the title and abstracts of 481 references were screened. Of these, 454 records were discarded, and 25 full-text articles were assessed. Fifteen studies fulfilled our eligibility criteria and were included. (Fig. 1 – PRISMA [Preferred Reporting Items for Systematic Reviews and Meta-analysis] Flowchart) [41]. Four of the selected papers were case-control studies [15, 26–28], one transversal [19], whereas nine were cohort studies [5, 16–18, 21–25, 42] (See: Table 1 Characteristics of included studies).

### Characteristics of patients included studies

#### Sample size

In total, the 15 studies included 8541 participants, 2932 cases had undergone an AFG procedure after immediate breast reconstruction or delay, and 5609 controls that have not undergone AFG.

#### Participants’ age

The mean age was described in 14 out of 15 studies in Table 1 [5, 15–19, 21–27, 42]. Twelve studies did not show differences between age groups [5, 15–19, 21–27, 42]. Two studies reported significant differences between groups, with the AFG group having the youngest participants age than the control group [16, 21].

#### Surgical indication

The breast surgery indications were 77.8% (6642/8541) due to invasive breast carcinoma, 16.3% (1390/8541) to carcinoma in situ*,* and 5.9% (509/8541) to prophylactic reasons.

### Interventions

The studies included patients that underwent mastectomy or breast-conserving surgery (BCS) for breast cancer treatment, and the AFG procedure was performed either at the same time of immediate breast reconstruction or in a second time surgery. In 7 out of 15 studies, only mastectomy procedures were included [5, 15–18, 21, 22]; 2 out of 15 included only BCS [19, 23], and 6 out of 15 studies, the patients underwent either BCS or mastectomy [24–28, 42].

In 10 out of 15 studies, the technique used to perform the AFG was Coleman [15, 17–19, 22–24, 26, 28, 42]. In the remaining six studies, the AFG technique was not mentioned [5, 16, 21, 25, 27].

### Adjuvant therapy

Twelve out of fifteen studies described some information about adjuvant therapy treatment (chemotherapy, endocrine therapy, and radiotherapy) [5, 15–19, 21–26, 28, 42], and three studies did not provide any information on adjuvant treatment [5, 15–19, 21–28, 42].

The indication of adjuvant therapy was based on clinical practice guidelines. In seven out twelve, there is no difference in adjuvant treatment between groups [5, 15–19, 21–26, 28, 42]. In five studies, the adjuvant treatment is different in control and the treatment arms [21, 24, 42]. In Krastev et al., the number of patients receiving hormonal therapy was 40% (119) and 50% (151) in the AFG and control groups, respectively; (*P* = .01) [42]. In Kronowitz et al., the control group was more likely to have HER2/ neu-positive tumors (11.6% and 6.4%, respectively; *p* = 0.001) and more likely to receive chemotherapy (*p* < 0.001) [21]. Cases were more likely to receive hormonal therapy (*p* = 0.043). Sorrentino et al. chemotherapy was performed in 54.1% of AFG patients vs. 44.6% of control patients (*p* = 0.04) [24]. In Stumpf et al. mais quimioterapia na intervençaão 53% versus 37.1 (the author didi not describe the *p* value) [23]. Coliandro 84.1% na intervenção versus 66.7 no control [18].

### Follow-up

Seven studies had a mean follow-up of 60 months or greater [15, 17, 22, 24–26, 42], five studies had a mean follow-up ranging from 40 and 60 months [5, 16, 21, 27, 28]; and three studies had a mean follow-up of less than 40 months [18, 19, 23]. Only Sorrentino [24] and Kronowitz [21] had a different mean time of follow-up between intervention and control groups with a longer time for the intervention group, as shown in Table 1.

### The methodological quality of the studies and publication bias

The methodological quality of the studies was evaluated using the Downs and Black instrument for adapted quality assessment. Nine studies [5, 16, 17, 21, 22, 25–27, 42] were considered to be ‘good’, whereas five studies [15, 18, 19, 23, 24] were considered ‘fair’ and one study [28] was considered poor. Additional file 2 Table 1 lists the risks of bias in each of the selected studies*.* The analysis showed no publication bias, with *P* = .635 in Egger’s test [34].

### Meta-analysis

#### Overall survival

**Fig. 2:**
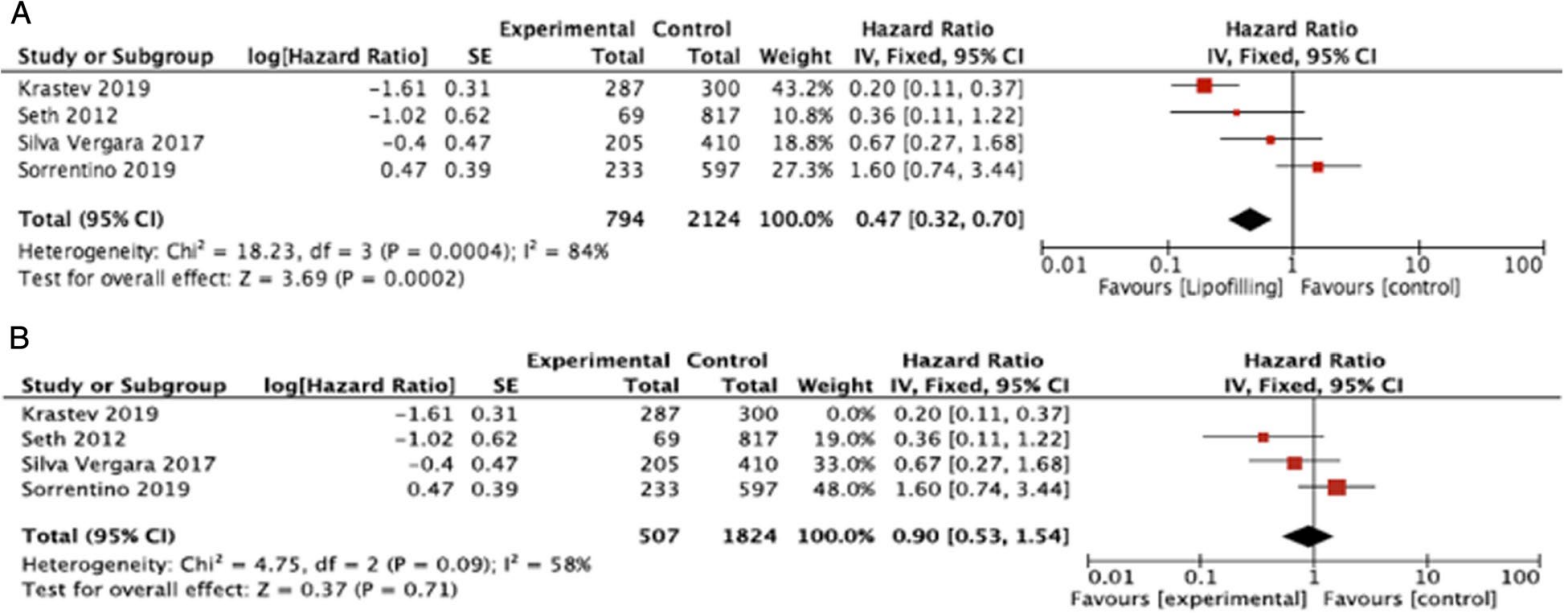
Forest plots demonstrating the results of the Overall Survival meta-analysis comparing AFG (lipofilling) versus control. **A** Shows the meta-analysis including the work of Krastev et al. [42]. **B** Shows the meta-analysis of the results after the exclusion of that manuscript due to the evidence of publication bias

**Fig. 3:**
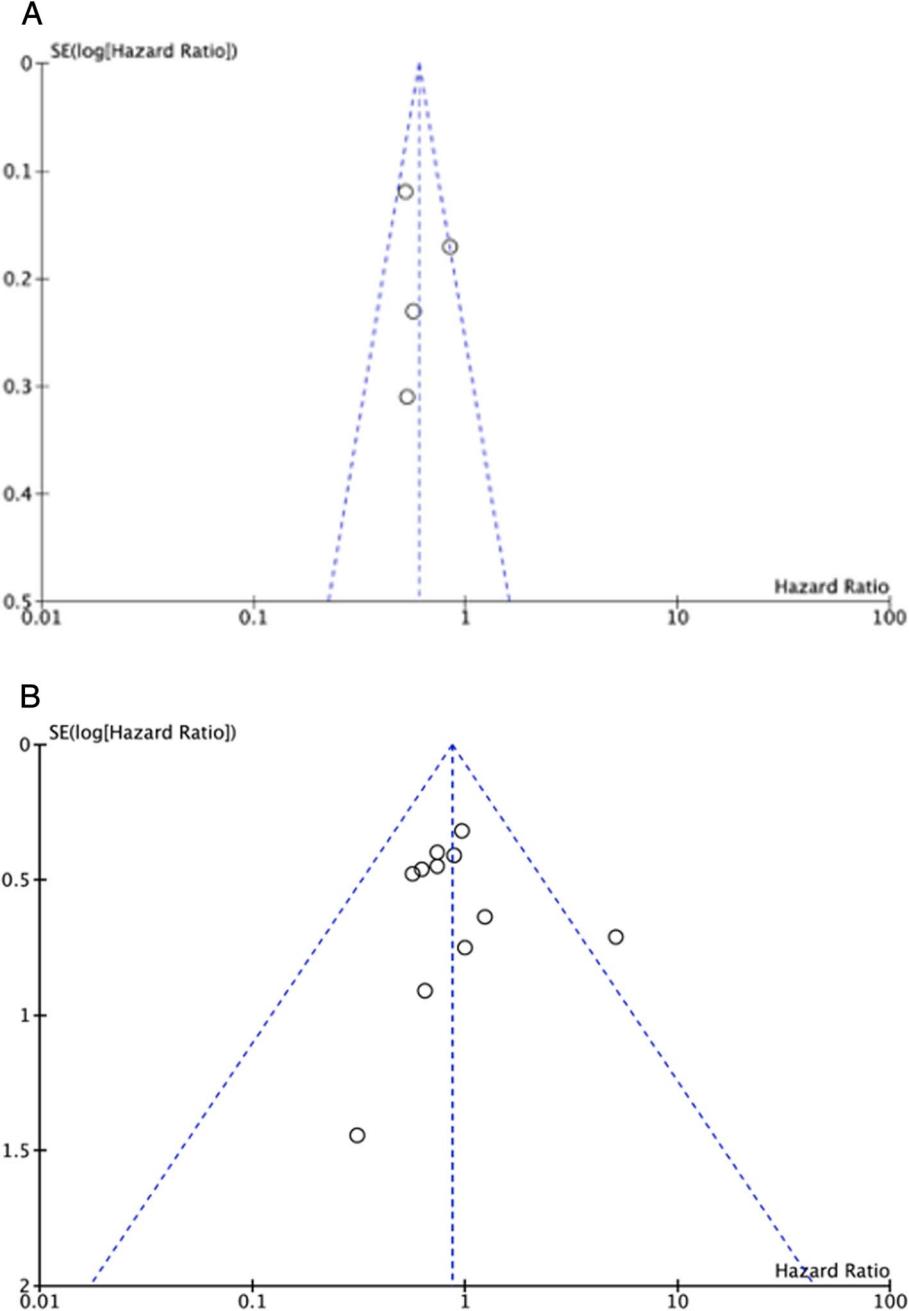
Funnel plots of the manuscripts included in the OS (**A**) and LR (**B**)

The data were not reported in sufficient detail for most studies, precluding hazard ratio (HR) calculation for overall survival outcome. The HR could be extracted from four studies [5, 24, 25, 42], and an increase of overall survival for the AFG group was detected with high heterogeneity (HR 0.47, 95% CI 0.32 to 0.7, *p* = 0.0002, four studies, 2918 participants, I^2^ = 84%, moderate; Fig. 2, Table 2). The funnel plot (Fig. 3A) indicated a high risk of publication bias from one study – Krastet al. al [42] which had higher mortality in the control group. The analysis excluding this article difference found no difference between AFG group and control, and publication bias was not detected (HR 0.9, 95% CI 0.53 to 1.54, *p* = 0.71, three studies, 2331 participants, I^2^ = 58%, moderate; Fig. 2).

**Table 2:** Summary of findings table

Summary of findings:						
**Adipose fat transfer compared to non for breast cancer surgery**						
**Patient or population:** breast cancer surgery **Setting:** Breast Reconstruction **Intervention:** Adipose fat transfer **Comparison:** non						
Outcomes	Anticipated absolute effects^*^ (95% CI)		Relative effect (95% CI)	№ of participants (studies)	Certainty of the evidence (GRADE)	Comments
	**Risk with non**	**Risk with Adipose fat transfer**				
Overall Survivall (OS) assessed with: Time to any death follow-up: range 36 months to 88.7 months	**Low**		**HR 0.47** (0.32 to 0.70) [Overall Survivall]	2918 (4 non-randomised studies)	⨁⨁⨁◯ Moderate^a^	
	967 per 1.000	**984 per 1.000** (977 to 989)				
Disease Free Survival (DFS) assessed with: time to any systemic or local recurrence event follow-up: range 36 months to 89 months	**Low**		**HR 0.90** (0.65 to 1.25) [Systemic or local progression]	2629 (7 non-randomised studies)	⨁⨁⨁◯ Moderate^b,c^	
	915 per 1.000	**923 per 1.000** (895 to 944)				
Local Recurrence (LR) assessed with: time to local recurrence (months) follow-up: range 36 months to 120 months	**Low**		**HR 0.87** (0.64 to 1.16) [Local Recurrence]	6713 (11 non-randomised studies)	⨁⨁⨁◯ Moderate^d,e^	
	970 per 1.000	**974 per 1.000** (966 to 981)				

*The risk in the intervention group (and its 95% confidence interval) is based on the assumed risk in the comparison group and the relative effect of the intervention (and its 95% CI)

*CI* confidence interval, *HR* hazard Ratio

GRADE Working Group grades of evidence

High certainty: we are very confident that the true effect lies close to that of the estimate of the effect

Moderate certainty: we are moderately confident in the effect estimate: the true effect is likely to be close to the estimate of the effect, but there is a possibility that it is substantially different

Low certainty: our confidence in the effect estimate is limited: the true effect may be substantially different from the estimate of the effect

Very low certainty: we have very little confidence in the effect estimate: the true effect is likely to be substantially different from the estimate of effect

Explanations

^a^ Krastev et al. which has higher mortality in the control group

^b^ Ferscth et al. - does not provide the estimatives of variability in the data for main outcomes, adverse effects, atrrition bias, patients were not representative of the target population, without adjustment for confounders

^c^ Stumpf et al. does not inform adverse effects, atrittion bias, without adjustment for potential confounders, does not have statistical power to detect difference

^d^ Mazur et al. does not inform adverse effects, atrittion bias, patients were not representative of the target population. Cases and controls were recruited from different populations. There is evidence of data dredging. There is no adjustment according to follow up. Inadequate statistical analysis. There is no statistical power to detect difference.

^e^ Petit et al. 2013 included only DCIS tumors

#### Disease-free survival

**Fig. 4:**
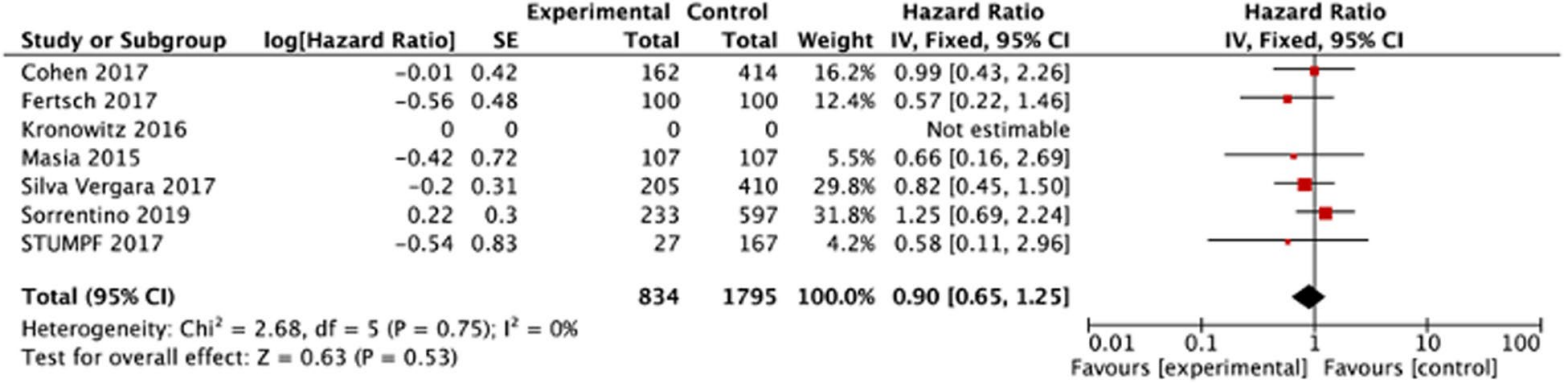
Forest plots demonstrating the results of the Disease-Free Survival meta-analysis comparing AFG (lipofilling) versus control

The HR could be extracted from seven studies [15, 16, 21–25] for DFS analysis, and no difference was found between the AFG group and control (HR 0.9, 95% CI 0.65 to 1.25, *p* = 0.53, seven studies, 2629 participants, I^2^ = 0%, moderate; Fig. 4, Table 2).

#### Local recurrence

**Fig. 5:**
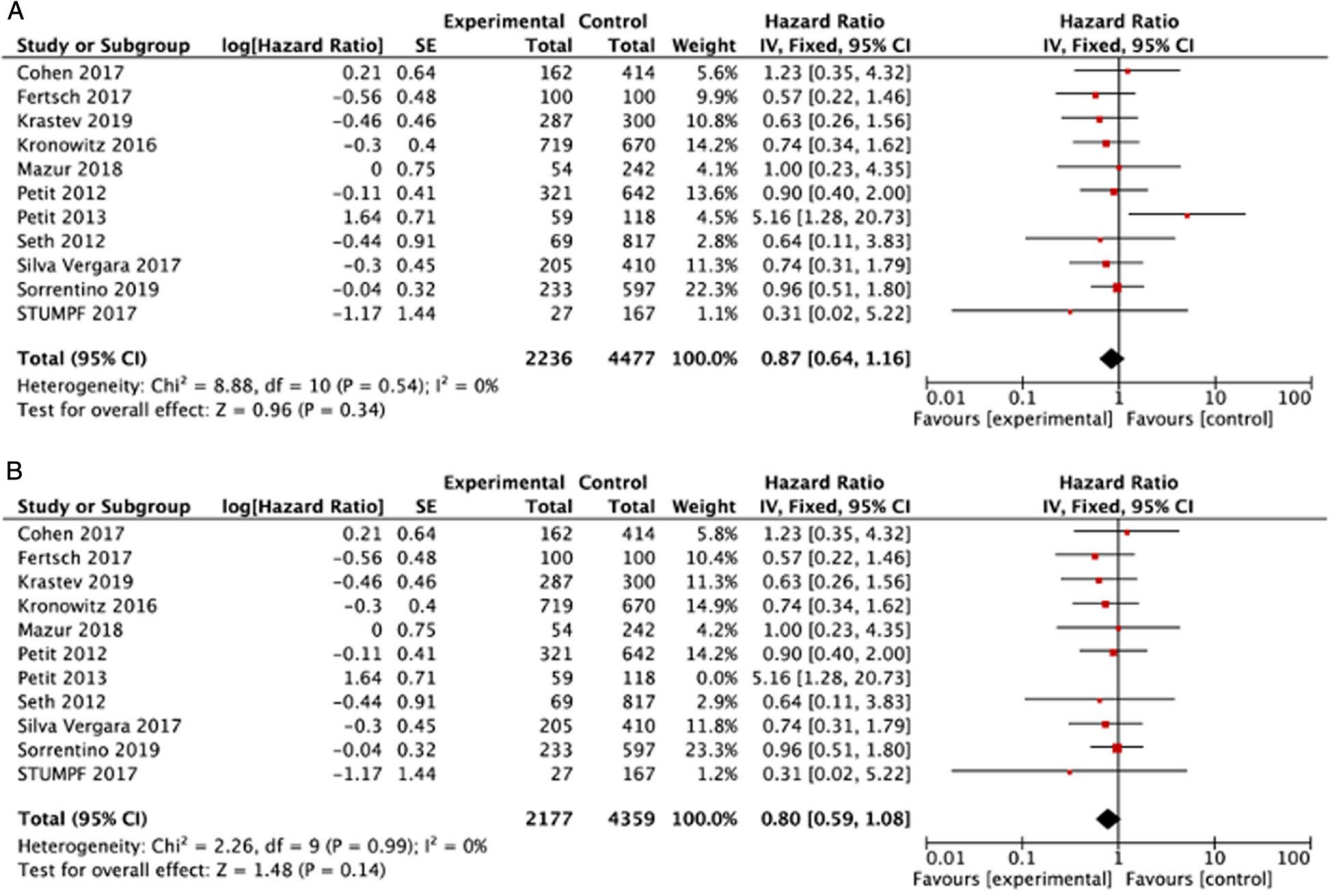
Forest plots demonstrating the results of the Local Recurrence meta-analysis comparing AFG (lipofilling) versus control. **A** Shows the meta-analysis including the work of Petit et al. from 2013 [26]. **B** Shows the meta-analysis of the results after the exclusion of that manuscript due to the evidence of publication bias

The HR could be extracted from eleven studies [5, 15, 16, 21, 23–28, 42] for local recurrence analysis and no difference was found between AFG group and control (HR 0.87, 95% CI 0.64 to 1.16, *p* = 0.34, 11 studies, 6713 participants, I^2^ = 0%, moderate; Fig. 5A, Table 2). The funnel plot (Fig. 3B) indicated possible publication bias from one study, Peet al.t al [26], that included only DCIS tumors. The analysis excluding this article did not demonstrate a difference in results between groups. (HR 0.8, 95% CI 0.59 to 1.08, *p* = 0.14, 10 studies, 6536 participants, I^2^ = 0%, moderate, Fig. 5B, Table 2).

## Discussion

Based on the results from this review, including 15 observational studies (prospective and retrospective cohort studies) evaluating the oncological safety of AFG in breast cancer patients, there were no differences regarding overall survival, disease-free survival, and local recurrence between patients who were subject to autologous fat grafting or not in breast reconstruction procedures.

For overall survival, this research provides a good indication of the likely effect; although only observational studies were includes, a considerable number of participants (2331 participants) contributed to the analysis, and all plausible confounders (biases) were well balanced between groups. The populations in the controll and intervention groups were similar. A follow-up period ranging from 42 to 86 months gives this results strength to conclude that autologous fat grafting is a safe procedure and should be indicated according to breast reconstruction surgeons’ evaluation without compromise oncological safety. The AFG technique was not described in only 2 of the 15 studies included in our analysis [21, 27]. In both studies, the authors found no difference in LRR or systemic recurrence rates between the AFG and control groups. These results are in accordance with the ones from the other studies included in this meta-analysis and do not interfere with the interpretation of our findings.

We could extract hazard ratios from seven studies comparing the autologous fat grafting or not in breast cancer treatment [15, 16, 21–25]. The confidence interval indicated no statistical difference in the disease-free survival in the intervention and in the control groups with 0% of heterogeneity, which agrees with published literature.

One of the goals of breast cancer surgical treatment is to reduce the risk of local recurrence. It is entirely established that when local recurrence usually occurs, it is followed by distant metastasis, reducing the overall survival of this group of patients [43–45]. This review showed that the time-to-local recurrence in breast cancer patients is not affected when the autologous fat grafting is part of the breast reconstruction procedure based on the results from those studies, including a significant number of participants [5, 15, 16, 21, 23–28, 42] with a low likelihood of residual confounding and a narrow confidence interval.

On a PubMed search, we identified 12 meta-analyses published evaluating AFG in breast cancer patients that underwent breast reconstruction [20, 46–56]. 4 of those evaluated complications associated with the procedure [48, 52, 53, 56], 2 evaluated aesthetical outcomes [34, 51], two evaluated different fat grafting techniques [54, 55] and only four evaluated local recurrence as an oncological outcome [46, 47, 49, 50]. None of these four that evaluated oncological outcomes had a formal evaluation of the quality of the studies, and none of them evaluated OS. These four studies presented the local recurrence as rates of the event instead of HR, which is the adequate metric for time-to-event outcomes. To our knowledge, this is the first meta-analysis to use this metric to assess the oncological safety of AFG.

This is the first systematic review with a meta-analysis of observational studies from this topic that evaluated the certainty of evidence through an appropriate tool (GRADE). A sensitive search strategy was carried out for all electronic databases, a manual search was made of the reference lists of relevant studies, and we screened clinical trial registries to avoid missing relevant studies. The methodological quality of the observational studies included was evaluated and considered in the presentation of our findings.

The strengths of the present study include our extensive search for pertinent AFG studies, the systematic application of eligibility criteria, the proper consideration of study quality, and our meticulous, analytical approach. However, the main limitations of this systematic review are the potential biases in the review process due to the methodological flaws of the included studies. The evidence in this review came from case-control studies; even though they were well planned, most of them were retrospective, which could overestimate the results. According to the GRADE evaluation, the true effect obtained from this meta-analysis is likely to be close to the estimate of the effect. However, there is a possibility that different results could be found if RCTs assessed this issue. Moreover, it was not possible to calculate the hazard ratio for the assessment of survival data for all studies because many of them did not report time-to-event analyses in sufficient detail. It is rightly emphasized that to carry out reviews, several subjective judgments are required, and a different review team might make slightly different decisions regarding the assessments of eligibility, risks of bias, and evaluated the certainty of evidence.

Even though randomized controlled clinical trials (RCTs) are considered the gold standard for evidence-based medicine due to their lower chances of selection bias and “confounding” effect, sometimes, they are not feasible, especially in the surgical field. This review showed an absence of randomized clinical trials to evaluate autologous fat grafting’s safety in the breast reconstruction field for breast cancer patients. However, the available observational studies did not whittle down our meta-analysis’s results; the studies included have great internal validity, with a high number of participants. In a scenario where an RCT is not feasible to perform, we would like to suggest important points for planning and conducting cohort studies in the surgical oncological field, trying to support this review update. The relevant issues are the CONSORT Statement to guide the manuscript writing, the objective definition of the assessed outcomes, methods for their measurement, and appropriate adjustment for follow-up. Based on this, it is crucial to analyze time-to-event outcomes using survival analysis methods, which were employed in our work, or person-years of follow-up as the denominator for the incidence rates for events of interest. However, prospective RCTs with adequate follow-up still have their established role in confirming the AFG oncological safety following breast cancer reconstruction definitively and further commend its safety concerning breast cancer detection and surveillance.

## Conclusion

The evidence found in this review is highly suggestive that AFG in breast cancer patients is a safe procedure based on data from 2331 patients included in 3 studies that contributed for OS analysis and 2629 patients included in 6 studies that contributed for DFS analysis. Even though the number of studies is small, the number of included patients is over two thousand, contributing to the robustness of our findings. This evidence is based on observational studies; most of them well planned and well designed to deal with major confounders leading to reliable results. Randomized studies in this field are pretty difficult to be executed because of the low number of oncological events such as death and local recurrence in breast cancer patients; moreover, the economic issues with planning, organize and execute a randomized study with long follow-up time associated with the paucity of funding resources make the execution of an RCT to evaluate surgical procedures almost prohibitive. Additional research is not likely to have a meaningful impact on the estimated effect observed in this review. We conclude that AFG is oncologically safe, and the decision to perform this procedure should be made according to the patients’ and the physicians’ values and preferences.

With evolving breast reconstruction strategies, breast and plastic surgeons and patients face significant challenges when evaluating surgical options. Incorporating the oncological outcomes of AFG modalities and presenting the safety results will considerably facilitate decision-making for all involved parties. The present study will contribute substantially to advancing evidence-based rehabilitation care of patients with breast cancer and candidates for reconstruction.

## Supplementary Information

**Additional file 1.**

**Additional file 2.**

**Additional file 3.**

## Data Availability

The datasets used and analyzed during the current study are available from the corresponding author on reasonable request.
