# Supplementary material for: The oncological safety of autologous fat grafting: a systematic review and meta-analysis
Source: BMC Cancer. 2022 Apr 11;22:391. doi: 10.1186/s12885-022-09485-5 (PMC9004160; doi:10.1186/s12885-022-09485-5)
Supplement: Supplementary file 1 — Additional file 1. [file 12885_2022_9485_MOESM1_ESM.docx]

Additional file 1

The search strategies used for each database are presented below.

Central (Cochrane)

#1 MeSH descriptor: [Breast Neoplasms] explode all trees

#2 breast cancer or breast neoplasm or breast adenocarcinoma or breast carcinoma or breast tumour or breast tumor

#3 #1 or #2

#4 MeSH descriptor: [Mastectomy, Segmental] explode all trees

#5 MeSH descriptor: [Mastectomy, Subcutaneous] explode all trees

#6 segmental mastectom* or subcutaneous mastectom* or breast conserving surger* or partial mastectom* or nipple-sparing mastectom* or areola-sparing mastectom* or local excision mastectom* or limited resection mastectom*

#7 Autologous fat grafting

#8 #4 or #5 or #6 and #7

#9 #3 and #8

Medline

| **Searches** |
| --- |
| 1. randomized controlled trial.pt. |
| 1. controlled clinical trial.pt. |
| 1. randomized.ab. |
| 1. placebo.ab. |
| 1. Clinical Trials as Topic/ |
| 1. randomly.ab. |
| 1. trial.ti. |
| 1. (crossover or cross-over).tw. |
| 1. Pragmatic Clinical Trials as Topic/ |
| 1. pragmatic clinical trial.pt. |
| 1. or/1-10 |
| 1. Case-Control Studies/ |
| 1. Control Groups/ |
| 1. Matched-Pair Analysis/ |
| 1. Retrospective Studies/ |
| 1. ((case* adj5 control*) or (case adj3 comparison*) or control group*).ti,ab. |
| 1. or/12-16 |
| 1. Cohort Studies/ |
| 1. Longitudinal Studies/ |
| 1. Follow-Up Studies/ |
| 1. Prospective Studies/ |
| 1. Retrospective Studies/ |
| 1. cohort.ti,ab. |
| 1. longitudinal.ti,ab. |
| 1. prospective.ti,ab. |
| 1. retrospective.ti,ab. |
| 1. or/18-26 |
| 1. exp Breast Neoplasms/ |
| 1. (breast cancer or breast neoplasm or breast carcinoma or breast tumour or breast tumor).mp. |
| 1. or/28-29 |
| 1. exp Mastectomy, Segmental/ |
| 1. exp Mastectomy, Subcutaneous/ |
| 1. segmental mastectom*.mp. |
| 1. subcutaneous mastectom*.mp. |
| 1. breast conserving surger*.mp. |
| 1. partial mastectom*.mp. |
| 1. nipple-sparing mastectom*.mp. |
| 1. areola-sparing mastectom*.mp. |
| 1. local excision mastectom*.mp. |
| 1. limited resection mastectom*.mp. |
| 1. (limited resection adj5 mastectom*).mp. |
| 1. or/31-41 |
| 1. and/30,42 |
| 1. Animals/ |
| 1. Humans/ |
| 1. 44 not 45 |
| 1. 43 not 46 |
| 1. and/11,47 |
| 1. and/17,47 |
| 1. and/11,47 |
| 1. 50 and autologous fat grafting |

EMBASE

#1 random* OR factorial* OR crossover* OR cross NEXT/1 over* OR placebo* OR (doubl* AND blind*) OR (singl* AND blind*) OR assign* OR allocat* OR volunteer* OR 'crossover procedure'/exp OR 'double blind procedure'/exp OR 'randomized controlled trial'/exp OR 'single blind procedure'/exp

#2 'case control study'/syn OR ('case control' OR 'case base' OR 'case matched' OR retrospective) NEXT/3 (analys* OR design* OR evaluation* OR research OR stud* OR survey* OR trial*)

#3 (cohort OR concurrent OR incidence OR longitudinal OR followup OR 'follow up' OR prospective OR retrospective) NEXT/1 (analys* OR design* OR evaluation* OR research OR stud* OR survey* OR trial*) OR 'prospective method'/exp OR 'retrospective study'/syn

#4 'breast neoplasms'/exp OR 'breast neoplasms' OR 'breast cancer'/exp OR 'breast cancer' OR 'breast carcinoma'/exp OR 'breast carcinoma' OR 'breast tumour' OR 'breast tumor'/exp OR 'breast tumor'

#5 'segmental mastectomy'/exp OR 'segmental mastectomy'

#6 'subcutaneous mastectomy'/exp OR 'subcutaneous mastectomy'

#7 'breast conserving surgery'/exp OR 'breast conserving surgery'

#8 'partial mastectomy'/exp OR 'partial mastectomy'

#9 'nipple-sparing mastectomy'

#10 'areola-sparing mastectomy'

#11 'local excision mastectomy'

#12 'limited resection mastectomy'

#13 'limited resection' NEAR/5 mastectom*

#14 #5 OR #6 OR #7 OR #8 OR #9 OR #10 OR #11 OR #12 OR #13

#15 #4 AND #14

#16 #15 AND [humans]/lim AND [embase]/lim

#17 #1 AND #16

#18 #2 AND #16

#19 #3 AND #16

#20 # AND autologous fat grafting

LILACS

| #1(Mastectomia Segmentar) OR (Mastectomia Segmental ) OR (Mastectomy, Segmental ) OR (Lumpectomy) OR (Partial Mastectomy) OR (Breast-Conserving Surgery) OR (Ex E04.466.701) |
| --- |
| #2(Mastectomy, Subcutaneous) OR (Mastectomia Subcutânea) OR (Mastectomia Subcutânea) OR (Ex E04.466.823) |
| #3(Breast Neoplasms) OR (Neoplasias de la Mama) OR (Neoplasias da Mama) OR (Cancer of Breast) OR (Breast Cancer) OR (Breast Tumors) |
| #4 #1 OR #2 |
| #5 #3 AND #4  #6 #5 AND autologous fat grafting |
