# Supplementary material for: The oncological safety of autologous fat grafting: a systematic review and meta-analysis
Source: BMC Cancer. 2022 Apr 11;22:391. doi: 10.1186/s12885-022-09485-5 (PMC9004160; doi:10.1186/s12885-022-09485-5)
Supplement: Supplementary file 2 — Additional file 2. [file 12885_2022_9485_MOESM2_ESM.docx]

**The oncological safety of autologous fat grafting: a systematic review and meta-analysis**

Rodrigo Goncalves^1*#^, Bruna Salani Mota^1*^, Bruno Sobreira-Lima^1^, Marcos Desidério Ricci^1^, José Maria Soares Jr^2^, Alexandre Mendonça Munhoz^3^, Edmund Chada Baracat^2^, José Roberto Filassi^1^

Affiliations:

1. Setor de Mastologia da Disciplina de Ginecologia do Departamento de Obstetrícia e Ginecologia, Hospital das Clínicas da Faculdade de Medicina da Universidade de São Paulo, São Paulo, Brazil

2. Disciplina de Ginecologia do Departamento de Obstetrícia e Ginecologia, Hospital das Clínicas da Faculdade de Medicina da Universidade de São Paulo, São Paulo, Brazil

3. Disciplina de Cirurgia Plástica, Hospital das Clínicas da Faculdade de Medicina da Universidade de São Paulo, São Paulo, Brazil. Instituto de Ensino e Pesquisa Hospital Sírio-Libanes, São Paulo, Brazil.

*. These authors contributed equally towards the planning and execution of this manuscript

#. Corresponding author: Rodrigo Goncalves, e-mail: [rodgon82@yahoo.com](mailto:rodgon82@yahoo.com), phone: +55 11 96576-7337, Address: Avenida Dr. Arnaldo, 251, São Paulo, SP – CEP 01246-000, Secretaria Cirúrgica, 4o andar.

Running head: Meta-analysis on lipofilling safety

| **STUDIES**  **Year of Publication** | **Fertsch**  **2019** | **Cohen**  **2017** | | | **Calabrese**  **2018** | | **Cogliandro 2017** | | **Khan**  **2017** | | **Krastev**  **2019** | | **Kronowitz**  **2015** | | **Masia**  **2015** | | **Mazur**  **2018** | | **Petit**  **2012** | | **Petit**  **2013** | | **Seth**  **2012** | | **Silva-Vergara**  **2017** | | **Sorrentino**  **2019** | | **Stumpf**  **2017** | |
| --- | --- | --- | --- | --- | --- | --- | --- | --- | --- | --- | --- | --- | --- | --- | --- | --- | --- | --- | --- | --- | --- | --- | --- | --- | --- | --- | --- | --- | --- | --- |
| **Reporting** |  |  | | |  | |  | |  | |  | |  | |  | |  | |  | |  | |  | |  | |  | |  | |
| Is the hypothesis/aim/objective of the study clearly described? | 1 | 1 | | | 1 | | 1 | | 1 | | 1 | | 1 | | 1 | | 1 | | 1 | | 1 | | 1 | | 1 | | 1 | | 1 | |
| Are the main outcomes to be measured clearly described Introduction or Methods section? | 1 | 1 | | | 1 | | 1 | | 1 | | 1 | | 1 | | 1 | | 1 | | 1 | | 1 | | 1 | | 1 | | 1 | | 1 | |
| Are the characteristics of the patients included in the study clearly described? | 1 | 0 | | | 1 | | 1 | | 1 | | 1 | | 1 | | 1 | | 1 | | 1 | | 1 | | 1 | | 1 | | 1 | | 1 | |
| Are the interventions of interest clearly de- scribed? | 1 | 1 | | | 1 | | 1 | | 1 | | 1 | | 1 | | 1 | | 1 | | 1 | | 1 | | 1 | | 1 | | 1 | | 1 | |
| Are the distributions of principal confounders in each group of subjects to be compared clearly described? | 2 | 2 | | | 2 | | 0 | | 0 | | 2 | | 2 | | 2 | | 2 | | 2 | | 2 | | 2 | | 2 | | 2 | | 2 | |
| Are the main findings of the study clearly described? | 1 | 1 | | | 1 | | 1 | | 1 | | 1 | | 1 | | 1 | | 1 | | 1 | | 1 | | 1 | | 1 | | 1 | | 1 | |
| Does the study provide estimates of the random variability in the data for the main outcomes? | 0 | 1 | | | 1 | | 1 | | 1 | | 1 | | 1 | | 1 | | 1 | | 1 | | 1 | | 1 | | 1 | | 1 | | 1 | |
| Have all important adverse events that may be a consequence of the intervention been reported? | 0 | 1 | | | 0 | | 1 | | 0 | | 0 | | 0 | | 0 | | 0 | | 1 | | 0 | | 1 | | 0 | | 1 | | 0 | |
| Have the characteristics of patients lost to follow-up been described? | 0 | 1 | | | 0 | | 1 | | 0 | | 1 | | 1 | | 0 | | 0 | | 0 | | 1 | | 0 | | 1 | | 1 | | 0 | |
| Have actual probability values been reported for the main outcomes except where the probability value is less than 0.001? | 1 | 1 | | | 1 | | 1 | | 1 | | 1 | | 1 | | 1 | | 1 | | 1 | | 1 | | 1 | | 1 | | 1 | | 1 | |
| **External validity** |  |  | | |  | |  | |  | |  | |  | |  | |  | |  | |  | |  | |  | |  | |  | |
| Were the subjects asked to participate in the study representative of the entire population from which they were recruited? | 1 | 0 | | | 1 | | 0 | | 0 | | 1 | | 1 | | 1 | | 0 | | 1 | | 1 | | 1 | | 1 | | 1 | | 1 | |
| Were those subjects who were prepared to participate representative of the entire population from which they were recruited? | 0 | 0 | | | 1 | | 0 | | 0 | | 1 | | 1 | | 1 | | 0 | | 1 | | 1 | | 1 | | 1 | | 1 | | 1 | |
| Were the stay, places, and facilities where the patients were treated, representative of the treatment the majority of patients receive? | 1 | 1 | | | 1 | | 1 | | 1 | | 1 | | 1 | | 1 | | 1 | | 1 | | 1 | | 1 | | 1 | | 1 | | 1 | |
| **Internal validity - bias** |  |  | | |  | |  | |  | |  | |  | |  | |  | |  | |  | |  | |  | |  | |  | |
| Was an attempt made to blind study subjects to the intervention they have received? | 0 | 0 | | | 0 | | 0 | | 0 | | 0 | | 0 | | 0 | | 0 | | 0 | | 0 | | 0 | | 0 | | 0 | | 0 | |
| Was an attempt made to blind those measuring the main outcomes of the intervention? | 0 | 0 | | | 0 | | 0 | | 0 | | 0 | | 0 | | 0 | | 0 | | 0 | | 0 | | 0 | | 0 | | 0 | | 0 | |
| If any of the results of the study were based on “data dredging”, was this made clear? | 1 | 1 | | | 1 | | 1 | | 1 | | 1 | | 1 | | 1 | | 0 | | 1 | | 0 | | 1 | | 1 | | 1 | | 1 | |
| In trials and cohort studies, do the analyses adjust for different lengths of follow-up of patients, or in case-control studies, is the time period between the intervention and outcome the same for cases and controls? | 1 | 1 | | | 1 | | 1 | | 1 | | 1 | | 1 | | 1 | | 0 | | 1 | | 1 | | 1 | | 1 | | 1 | | 1 | |
| Were the statistical tests used to assess the main outcomes appropriate? | 1 | 1 | | | 1 | | 1 | | 1 | | 1 | | 1 | | 1 | | 0 | | 1 | | 1 | | 1 | | 1 | | 1 | | 1 | |
| Was compliance with the intervention/s reliable? | 1 | 1 | | | 0 | | 1 | | 1 | | 1 | | 1 | | 1 | | 1 | | 1 | | 1 | | 1 | | 1 | | 1 | | 1 | |
| Were the main outcome measures used accurate (valid and reliable)? | 1 | 1 | | | 1 | | 1 | | 1 | | 1 | | 1 | | 1 | | 0 | | 1 | | 1 | | 1 | | 1 | | 1 | | 1 | |
| **Internal validity - confounding (selection bias)** |  |  | | |  | |  | |  | |  | |  | |  | |  | |  | |  | |  | |  | |  | |  | |
| Were the patients in different intervention groups (trials and cohort studies) or were the cases and controls (case-control studies) recruited from the same population? | 1 | 1 | | | 1 | | 1 | | 1 | | 1 | | 1 | | 1 | | 0 | | 1 | | 1 | | 1 | | 1 | | 1 | | 1 | |
| Were study subjects in different intervention groups (trials and cohort studies) or were the cases and controls (case-control studies) recruited over the same period of time? | 1 | 1 | | | 1 | | 1 | | 1 | | 1 | | 1 | | 1 | | 0 | | 1 | | 1 | | 1 | | 1 | | 1 | | 1 | |
| Were study subjects randomised to intervention groups? | 0 | 0 | | | 0 | | 0 | | 0 | | 0 | | 0 | | 0 | | 0 | | 0 | | 0 | | 0 | | 1 | | 0 | | 0 | |
| Was the randomised intervention assignment concealed from both patients and health care staff until recruitment was complete and irrevocable? | 0 | 0 | | | 0 | | 0 | | 0 | | 0 | | 0 | | 0 | | 0 | | 0 | | 0 | | 0 | | 0 | | 0 | | 0 | |
| Was there adequate adjustment for confounding in the analyses from which the main findings were drawn? | 0 | 1 | | | 1 | | 1 | | 1 | | 1 | | 1 | | 1 | | 0 | | 1 | | 1 | | 0 | | 1 | | 1 | | 0 | |
| Were losses of patients to follow-up taken into account? | 0 | 1 | | | 1 | | 1 | | 0 | | 1 | | 1 | | 1 | | 1 | | 1 | | 1 | | 0 | | 1 | | 1 | | 0 | |
| **Power** |  |  | | |  | |  | |  | |  | |  | |  | |  | |  | |  | |  | |  | |  | |  | |
| Did the study have sufficient power to detect a clinically important effect where the probability value for a difference being due to chance is less than 5%? | 1 | 0 | | | 0 | | 0 | | 0 | | 1 | | 0 | | 1 | | 0 | | 0 | | 0 | | 0 | | 0 | | 0 | | 0 | |
| **Final score** | 18 | | 20 | 20 | | 19 | | 16 | | 23 | | 22 | | 22 | | 12 | | 22 | | 20 | | 23 | | 23 | | 19 | | 15 | |  |
