# Supplementary material for: The oncological safety of autologous fat grafting: a systematic review and meta-analysis
Source: BMC Cancer. 2022 Apr 11;22:391. doi: 10.1186/s12885-022-09485-5 (PMC9004160; doi:10.1186/s12885-022-09485-5)

**The oncological safety of autologous fat grafting: a systematic review and meta-analysis**

Additional file Figure 1. Flowchart of the study selection process according to PRISMA guidelines


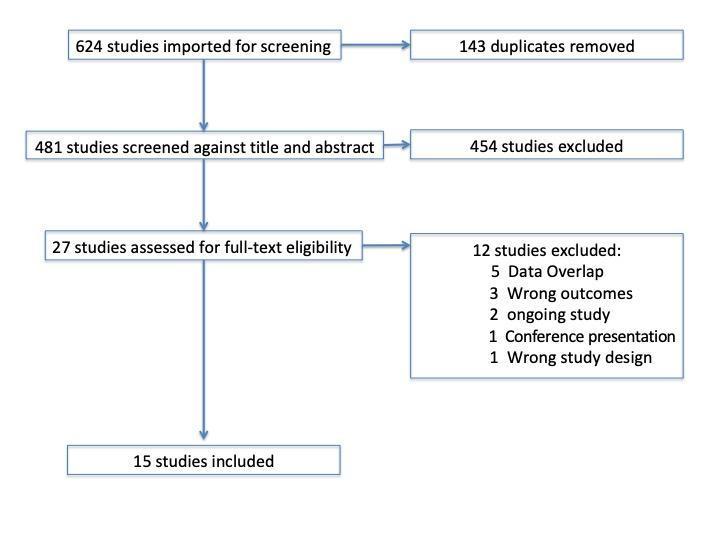

Supplement: Supplementary file 3 — Additional file 3. [file 12885_2022_9485_MOESM3_ESM.docx]
